# Deep learning and level set approach for liver and tumor segmentation from CT scans

**DOI:** 10.1002/acm2.13003

**Published:** 2020-08-10

**Authors:** Omar Ibrahim Alirr

**Affiliations:** ^1^ College of Engineering and Technology American University of the Middle East Kuwait Kuwait

**Keywords:** automatic segmentation, CT, deep learning, liver tumors, region‐based level set

## Abstract

**Purpose:**

Segmentation of liver organ and tumors from computed tomography (CT) scans is an important task for hepatic surgical planning. Manual segmentation of liver and tumors is tedious, time‐consuming, and biased to the clinician experience. Therefore, automatic segmentation of liver and tumors is highly desirable. It would improve the surgical planning treatments and follow‐up assessment.

**Method:**

This work presented the development of an automatic method for liver and tumor segmentation from CT scans. The proposed method was based on fully convolutional neural (FCN) network with region‐based level set function. The framework starts to segment the liver organ from CT scan, which is followed by a step to segment tumors inside the liver envelope. The fully convolutional network is trained to predict the coarse liver/tumor segmentation, while the localized region‐based level aims to refine the predicted segmentation to find the correct final segmentation.

**Results:**

The effectiveness of the proposed method is validated against two publically available datasets, LiTS and IRCAD datasets. Dice scores for liver and tumor segmentation in IRCAD datasets are 95.2% and 76.1%, respectively, while for LiTS dataset are 95.6% and 70%, respectively.

**Conclusion:**

The proposed method succeeded to segment liver and tumors in heterogeneous CT scans from different scanners, as in IRCAD dataset, which proved its ability for generalization and be promising tool for automatic analysis of liver and its tumors in clinical routine.

## 1 INTRODUCTION

Computer‐aided diagnosis and surgery system (CAD/CAS) for liver cancer is a group of methods that use the computing technology to help in the preoperative planning, resection risk assessment, or actual surgery treatments. Liver cancer is one of the most frequent cancerous diseases that cause high number of deaths every year. Liver cancer treatments require accurate diagnosis and planning.^1^

Liver treatment options require accurate diagnosis, to assess the size and location of the tumors and arrive at the best clinical decision on the treatment. Medical imaging using computed tomography (CT) scans is the most commonly used imaging techniques for liver cancer diagnosis, since it gives accurate anatomical information about the abdominal organs in the human body.^2^ Liver organ and tumor segmentation from CT scans is an important step in the visualization of liver anatomy for surgical planning.^3^

Manual segmentation of liver organ and tumors from CT scans is tedious and time‐consuming. It greatly depends on the skills of the physician or doctor who performs the segmentation task. Liver organ has high variability in terms of shape and volume between different patients.^4^ Low contrast and blurry edges are the main characteristics of CT images, which make liver delineation a challenging task.^5^ Tumor segmentation adds more challenge due to the small observable changes between tumor and healthy tissues especially at their borders. In addition, tumors vary greatly in terms of shape, size, and texture. Despite these challenges, which complicate tumor segmentation, the automated approach is desirable, as it is, ideally, more objective and removes dependence on human skill.^6^

Liver surgical planning treatments would benefit from an accurate and fast liver and tumor segmentation that allows for subsequent determination of tumor burden and texture‐based information. Moreover, having a standardized and automatic segmentation method would facilitate a more reliable therapy response classification.^7^

Organ segmentation from CT scans has been a hot research topic during the past few years. Recently, due to the advancement in computer vision, the development of deep fully convolutional neural (FCNs) networks enhanced the performance of the semantic segmentation, and leads to outperform other competitors in the field of medical imaging.^8^, ^9^ General FCN focuses its task on image classification, where input is an image and output is one label. However, in medical imaging, it requires, besides the classification, to localize the area of abnormality.^10^

Following the FCN success, many attempts have been carried to use the FCN for liver and tumor segmentation^11^; one of the best FCN architectures has been created is the U‐Net.^12^ U‐Net is succeeded to classify the images and locate the specific structures, and it has the ability to locate and distinguish borders by doing classification on every pixel.

Recently, the liver tumor segmentation (LiTS) competition challenge was organized in conjunction with ISBI 2017 conference.^13^ The top‐rated automatic methods submitted to the competition used FCN networks. For this purpose, different works used U‐Net architecture for liver and tumor segmentation.^11^, ^14^, ^15^

Despite the high accuracy achieved by deep learning FCNs in segmenting organs from CT scans, these methods depend on the training step on many datasets to cover all expected features of the intended organ and build a trained network to detect that organ in the test dataset. However, these methods overlook to get benefit of the local features in the test dataset itself to refine and improve the final segmentation from the target CT scan.

Due to the previously explained issue, the recent research moves toward the combination between deep learning methods with local information‐based techniques. In liver and tumor segmentation domain, many intensity‐based techniques have been proposed to find the intensity range of the liver and tumor by applying a statistical analysis on the intensities in CT scans.^16^, ^17^ One of these techniques that can be used more independently is the level set‐based active contour methods.^18^ Level set‐based active contour method is used to deform an initial mask, that is coarse segmentation, to match more accurately the boundary of the liver/tumor in the test CT scan.^19^, ^20^

In this work, two cascaded FCN networks are constructed using U‐Net, the same work proposed by Christ et al.^21^ The first subnetwork aims to locate and predict the liver organ, while the second subnetwork work on the segmented liver envelope to detect and segment tumors. The output of these networks represents the coarse segmentation of liver and tumors, that are considered as initial mask used by the level set method to be deformed to the liver/tumor boundaries in the target CT image and generate the final segmentation.

## 2 MATERIALS AND METHODS

### 2.A Overview of the proposed framework

**Fig. 1:**
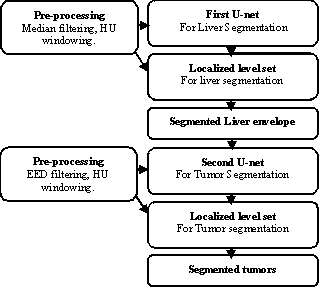
Overview of the proposed liver and tumor segmentation framework.

The proposed segmentation framework is presented in Fig. 1. The workflow consists of three main steps. It is applied first for liver segmentation and then for tumor segmentation. The first step (Section 2.B) deals with data preprocessing, windowing, and filtering steps are applied on LiTS datasets for liver and tumor segmentation. In a second step (Section 2.C), U‐Net FCN is constructed and trained—one network to segment liver organ and another network is trained to segment the tumors inside the liver region of interest (ROI). In the third step (Section 2.D), the localized level set is applied on the predicted U‐Net segmentation for further enhancement to get the final liver and tumor segmentation.

### 2.B Data preparation

**Fig. 2:**
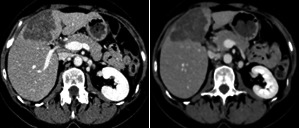
Effect of windowing and median filtering. The raw computed tomography slice (left) and the enhanced slice using median filtering (right).

For liver segmentation step, the contrast‐enhanced CT scans undergo median filtering to improve intensity homogeneity especially in liver region. Step of intensity windowing is applied to exclude irrelevant organs and focus on liver organ intensity range. Based on intensity‐based techniques,^16^, ^17^, ^18^, ^19^, ^20^ liver organ and tumors Hounsfield (HU) intensities range is 0–200. In this work, HU windowing is applied on datasets used in the FCN training step for both liver and tumor segmentations, the used HU window is −50–250. Figure 2 presents the effect of applying windowing and median filtering on CT slice example.

For tumor detection and segmentation using U‐Net network, the training datasets are enhanced using tensor‐based 3D edge enhancing diffusion (EED) filtering,^22^ that would improve the prediction of U‐Net network to detect and segment tumors. EED filtering uses diffusion tensor to adapt the diffusion based on the image structure. Edge enhancing diffusion filter is used to enhance the contrast, filters the noise in the homogeneous regions, and preserves the boundaries of the shape.^22^

**Fig. 3:**
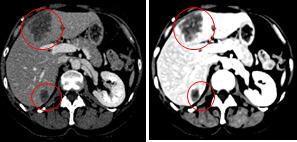
Computed tomography scan enhancement using edge enhancing diffusion filtering.

Edge enhancing diffusion filtering enhances the contrast of tumors by enhancing the homogeneities inside the liver and tumor tissue regions. In addition, it preserves the boundaries between tumors and liver tissue. Figure 3 shows an example of a CT scan before and after being enhanced using EED filtering. The intensity of the liver parenchyma is enhanced and appears brighter than the tumor regions, while the tumors appear darker compared to the liver tissue.

In this work, EED filtering improves the contrast of tumor structures by enhancing the homogeneities inside the liver and tumor tissue regions, and preserves the boundaries between them. This step would teach and orient the U‐Net FCN network to extract and learn the features that differentiate the tumor structure from the surrounding tissues. Figure 3 shows the effect of the preparation step on the raw medical CT slice.

### 2.C Fully convolutional neural networks

In this work, the U‐Net architecture is used to build the FCNs. The networks are used to compute the soft probability label maps. Both U‐Nets enable accurate pixel‐wise prediction by combining spatial and contextual information in a network architecture comprising 19 convolutional layers.

**Fig. 4:**
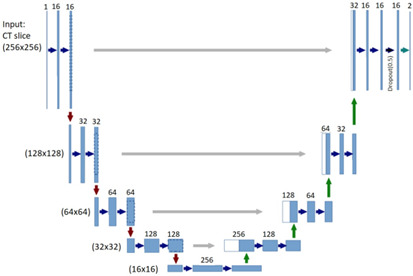
Illustration of U‐Net fully convolutional neural network architecture for liver/tumor segmentation.

Figure 4 shows the U‐Net architecture, the input passes and is processed by a sequence of convolution blocks, where the feature maps are doubled and resolutions are decreased (contracting path). The expanding path of the U‐Net reverses the process using the transposed convolution. The network contains dropout layer (0.5) before the final output layer to avoid over fitting. The output layer is designed using a linear classifier, sigmoid, that outputs a probability value (0–1) for each pixel being liver (tumor) or the background. The U‐Net FCN architecture is implemented using Keras^1^ with the TensorFlow backend.

The two U‐Net FCNs are implemented in a cascading way. 100 LiTS CT scans with various image dimensions are used for training. All training slices are resized to have common size, so the inputs for both FCNs are two‐dimensional (2D) grayscale slices of size 256 × 256, and their outputs are binary mask images of size 256 × 256.

The first network is trained to segment the liver envelope in whole abdomen slices, which are resampled to input size (256 × 256), so that the network concentrates on learning features that discriminate liver from background. The second network is trained to segment the tumors, given the liver envelope image. The segmented liver from the first framework step is cropped and resized to the second network input. The liver ROI helps in reducing the percentage of misclassified nontumor pixels. The second U‐Net FCN can concentrate on learning features that discriminate tumors from liver background segmentation.

The soft dice coefficient (DSC) is used as loss function that is computed on the pixel‐wise softmax of the FCN final feature map. Due to segmenting small objects like tumors, class balancing according to the pixel‐wise frequency of each class in the data is required. To deal with this case, the training datasets ensured to have the corresponding mask for each input 2D slice, so each batch contains patches where both tumor and background are present. In addition, to focus the model on the liver/tumor structure, the training process excludes slices that does not have corresponding mask.

**Fig. 5:**
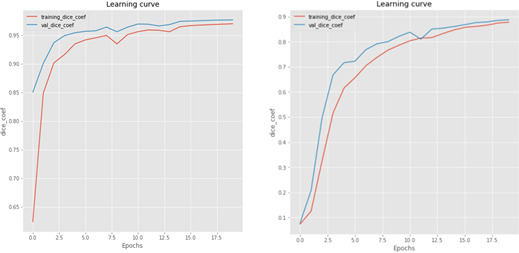
Learning curves of the two U‐Net fully convolutional neural networks: Liver U‐Net (left) and Tumors U‐Net (Right).

Both networks are trained with 20 epochs (mini‐batch size 32). The network parameters are updated using Adam optimizer with 0.001 learning rate. The learning rate is reduced by factor of 0.1 every 5 epochs to ensure a balanced loss, if no improvement in network optimization is acquired. Figure 5 shows the learning curves of the proposed FCNs for liver and tumors; the achieved validation accuracies for liver and tumor FCNs are 97.7% and 88.8%, respectively.

### 2.D Localized region‐based level set

Fully convolutional neural‐predicted segmentation may not reach the liver or tumor boundaries in some test CT scan, since the FCN output accuracy is limited by the learned features from the training datasets. Level set‐based active contour method is used in this work to refine the FCN‐predicted segmentation, to match more accurately the boundary of the liver organ or the tumors inside the CT scan.

Active contour‐based techniques have been widely used for image segmentation and boundary tracking.^18^ The basic idea of active contour methods is to start with initial boundary shapes represented in a form of closed curves, that is contours, and iteratively allow the contour to deform so as to minimize a given energy functional according to the constraints of the image, in order to produce the desired segmentation. Level set‐based active contour is a formulation to implement active contours that was proposed by Osher and Sethian.^23^

Two main categories exist for level set active contours: edge‐based and region‐based. Edge‐based active contour models utilize image gradients in order to identify object boundaries; however, this type has been found to be very sensitive to image noise and depend on the initial contour place. On the other hand, the region‐based level set active contour has advantages compared to edge‐based level set methods that include robustness against initial contour place and insensitivity to image noise.^24^

Since the FCN‐predicted segmentation is expected to be close to the liver/tumor boundary, the region‐based level set active contour seems to be more suitable than other level set types, namely that proposed by Chan and Vese.^18^ The Chan‐Vese energy (Ecv), which is aimed to be minimized, is referred by Eq. (1):

(1) $$E_{\mathrm{CV}}\left[C\right]=\mu \int_{0}^{L\left(C\right)}\mathrm{ds}\;+\iint_{\Omega_{c}}{\left(I\left(x,y\right)‐c_{1}\right)}^{2}dxdy\;+\iint_{\Omega_{c}}{\left(I\left(x,y\right)‐c_{2}\right)}^{2}dxdy$$

where Ωc represents the interior of the curve C, and c1 and c2 are the mean intensities for the interior and the exterior of the curve to be defined in an image I. The first term is the regularization term that minimizes the curve length s, and the second term maintains the balancing between the interior and the exterior. To make this step more efficient, the localized implementation of this active contour method is used.^25^

**Fig. 6:**
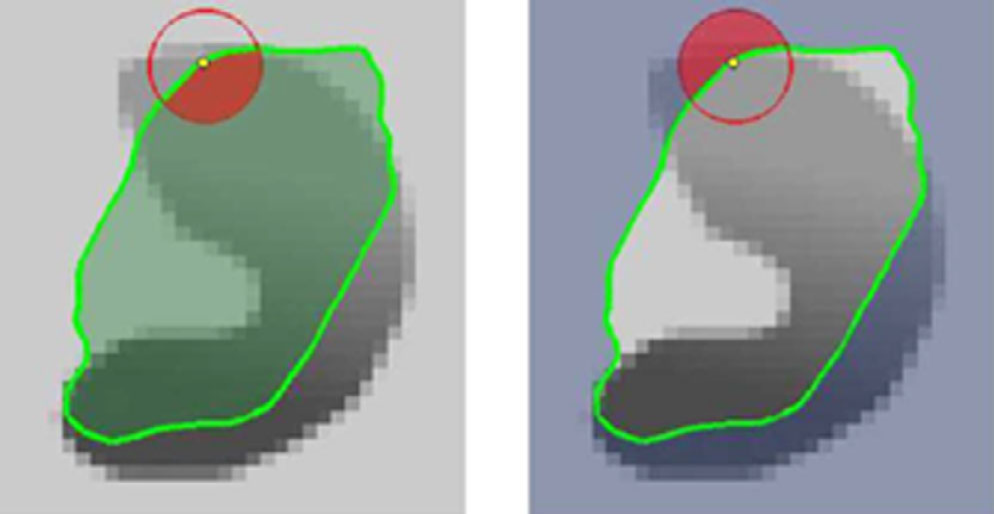
Local interior and local exterior regions.^25^

Instead of modeling the region of the whole image, the curve is modeled by many neighborhood local regions, each local region is considered separately, which is divided into local interior and local exterior, as explained in Fig. 6. After defining the interior and exterior local regions, the energy optimization then done by fitting the curve at each local region, the algorithm incorporates the Chan‐Vese energy implementation^18^ to model the local interior and exterior forces of each local region contour.

The localized active contour step is used in both liver and tumor segmentation steps. The FCN‐predicted output is used as an initial contour (C, in Eq. 1) for the level set function, that is the coarse liver or tumor as initial segmentation for the liver or the tumor segmentation steps.

Starting from U‐Net‐predicted segmentation of the liver or tumor, the localized contouring algorithm is applied on all slices sequentially. Besides the HU windowing, a simple thresholding step using Otsu technique is applied to extract and concentrate more on the liver intensity range for the liver segmentation step. The same thresholding is applied on the EED‐enhanced liver ROI to extract more accurate tumor intensity range inside the liver envelope. The thresholded intensities help the localized level set function to deform the initial FCN segmentation with limited number of iterations to match the liver/tumor boundaries and generate best final segmentation.

## 3 RESULTS

### 3.A Datasets

The FCN networks are trained using a subset of publically available training LiTS datasets. LiTS training datasets^2^ contain 131 contrast‐enhanced CT scans obtained from different clinical institutions. The CT scans come with manual delineation of the liver and tumors done by experts. The LiTS dataset contains 908 lesions.

The LiTS datasets are divided into two groups, 100 datasets used for FCN training and validation, while the remaining 31 datasets are used for testing and evaluation. For LiTS datasets that come without tumor masks, they are excluded from the FCN training step for tumor segmentation. The number of slices extracted from the 100 LiTS datasets that are used for FCN network training are 15 125 and 3459 for liver and tumors segmentation, respectively.

In order to demonstrate the robustness, generalization, and scalability of the proposed method, the proposed method is applied on 50 datasets from two publically available datasets, LiTS and IRCAD. As mentioned earlier, 31 LiTS CT scans are devoted for testing and evaluation. Besides that, the 3D IRCAD dataset^3^ is also used for testing and evaluating the proposed methods. IRCAD dataset has higher variety and complexity of livers and its tumors, and IRCAD dataset includes 20 venous phase‐enhanced CT volumes acquired with different CT scanners. IRCAD datasets are pathological CT cases, which have 111 tumor cases residing inside the liver envelope.

### 3.B Qualitative and quantitative results

**Fig. 7:**
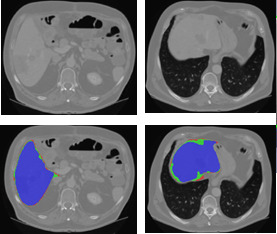
Automatic liver segmentation with fully convolutional neural networks U‐Net and region‐based level set. Green depicts correct liver segmentation (level set + U‐Net). Blue for predicted liver segmentation (using U‐Net) and red is the corresponding ground truth.

The qualitative results of the automatic liver segmentation for two different examples are visualized in Fig. 7. Comparison with segmented liver using the proposed method, U‐Net‐predicted segmentation and ground truth, gives rise to the assumption that the proposed approach is highly promising to achieve high performance.

The U‐Net computes the soft label probability maps. It examines each pixel of the test CT scan and assigns it to one of the two labels, liver or background for the liver segmentation step, and tumor or liver tissue for tumor segmentation step. The localized region‐based level set step deforms the U‐Net output (liver or tumor) to match the boundary of the structure based on the intensity differences around the initial contour.

The proposed method succeeded to segment the liver organ from different CT scans that come with complex structures and different intensity homogeneities. In general, it can be observed from Fig. 7 the significant improvement of the localized level set step in enhancing the U‐Net‐predicted segmentation. It deforms the FCN output segmentation (blue) to match the liver boundary (green) with high accuracy in the target CT scan.

**Fig. 8:**
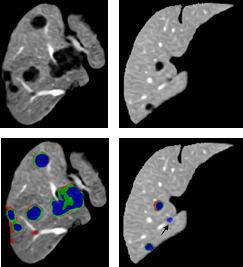
Automatic tumor segmentation from liver region of interest with U‐Net FCN and region‐based level set. Green depicts correct tumor segmentation (level set + U‐Net), blue for predicted liver segmentation (using U‐Net) and red is the corresponding ground truth.

Figure 8 shows the qualitative results of the proposed approach for automatic tumor segmentation inside liver ROI for different examples. The examples in the figure highlight the differences by comparing the segmented tumor (green) using the proposed method (level set + U‐Net) and U‐Net‐predicted segmentation (blue) with ground truth segmentation (red).

For tumor segmentation, the level set improvement on the U‐Net‐predicted segmentation is relatively small compared to liver segmentation, as the U‐Net succeeded with high accuracy to learn and segment the tumor from the EED‐enhanced liver ROI. However, the other side of level set improvement on final tumor segmentation is to exclude nontumorous objects that could appear in the predicted segmentation, as pointed by the arrow in the second example of Fig. 8. The level set method aims to maintain mean intensities on both contour sides, which does not work with nontumor small objects. These structures contain similar mean intensities inside and outside the contour that would lead the contour to collapse, vanish, and not appear in the level set segmentation.

To quantitatively evaluate the proposed performance, the most commonly used evaluation metric in semantic image segmentation, dice coefficient (DSC), is applied. The DSC, defined by (2), is an overlap measure that computes the ratio between the correctly segmented pixels (intersection) with respect to the average number of voxels of the segmentation output and the ground truth. Where (A) denotes the segmentation result by the proposed method and (B) is the ground truth segmentation.

(2) $$\text{Dice}\;\left(A,B\right)=2*\left|A\cap B\right|/\left(\left|A\right|+\left|B\right|\right).$$

The two FCNs for liver and tumor segmentation predictions are trained using 100 LiTS datasets with Dice metric as training accuracy metric. The achieved validation accuracies for liver and tumor FCNs are 97.7% and 88.8%, respectively.

The Dice (DSC) per case evaluation metric values for liver organ segmentation step is summarized in Table 1. The table shows the mean Dice results for both LiTS and IRCAD evaluation datasets. In addition, the table presents the improvement of level set steps on the predicted FCN output. The liver segmentation evaluation scores demonstrate that the proposed method performs remarkable good, provided that the combined level set and U‐Net approach outperforms the use of only U‐Net FCN, and the use of level set boosted the liver segmentation performance.

**Table 1:** Mean dice evaluation for automatic liver segmentation.

Method	IRCAD	LiTS
U‐Net FCN	88.1%	90.2%
U‐Net + level set	95.2%	95.6%

The Dice values for liver tumor segmentation step are summarized in Table 2. The table shows the mean Dice per case results for both LiTS and IRCAD evaluation datasets. In addition, the table highlights the level set improvement on the predicted FCN output, which is relatively small compared with liver segmentation step. This is due to the small size of tumor segmentation compared to liver organ segmentation. In addition, the FCN network improvement is high because it succeeded to learn and extract the tumor features from EED‐enhanced training datasets that helped in predicting the tumors’ structure inside the target liver ROI.

**Table 2:** Mean dice evaluation for automatic tumor segmentation.

Method	IRCAD	LiTS
U‐Net FCN	75.0%	64.7%
U‐Net + level set	76.1%	70.0%

The mean Dice values for LiTS dataset are lower than for IRCAD dataset, due to the reason that LiTS set contains several very small tumors that make it hard for FCN and level set to detect and segment them. On other side, the level set improvement on FCN output for LiTS dataset is larger compared to the IRCAD, since the level set step helped in removing the small nontumorous predicted objects from the FCN output. Moreover, Table 2 demonstrates that while the FCN U‐Net is trained using LiTS datasets without any fine‐tuning toward IRCAD datasets, the method shows impressive results for IRCAD segmentation. Hence, the proposed method demonstrates strong generalization and scalability features.

Table 3 presents the proposed method listed with other related works that implement 2D FCN network to segment liver and tumors from CT scans. Most of these methods use U‐Net implementation or deviated version of U‐Net, to design their models.^15^ The table outlines the related work performance in terms of Dice metric values for LiTS and IRCAD datasets. The proposed method has a comparable performance in terms of liver segmentation compared to other works, while it shows a superior performance for tumors segmentation.

**Table 3:** Dice comparison of the liver and tumor segmentation on LiTS and IRCAD datasets.

Method (dataset)	Liver	Tumor
Chen et al.^29^ (LiTS)	95.7%	66.6%
Ahmad et al.^26^ (IRCAD)	91.8%	–
Bi et al.^30^ (LiTS)	93.4%	64.5%
Christ et al.^21^ (IRCAD)	94.3%	56.0%
Vorontsov et al.^15^ (LiTS)	95.1%	66.1%
Yuan et al.^28^ (LiTS)	96.3%	65.7%
Nanda et al.^31^ (LiTS)	95.5%	69.7%
Chlebus et al.^11^ (LiTS)	96.0%	67.6%
Proposed method (IRCAD)	95.2%	76.1%
Proposed method (LiTS)	95.6%	70.0%

The proposed method outperforms other similar works that rely on combining local‐based techniques with FCN. Some works combined level set with FCN^26^; however, they used different form of U‐Net that examines blocks of image features instead of pixel‐wise in network learning, and they used level set as refining postprocessing step. Other method that was introduced by Nanda et al utilizes cascaded U‐Net networks, which are optimized using genetic algorithm (GA), to segment liver and tumors from LiTS dataset. Other authors proposed a model that consists of two U‐Nets that are connected and trained sequentially in one step from end to end, and simultaneously segmented into three classes.^15^ However, the one‐step model approach could work fast but is prone to misclassification, especially in the tumor segmentation task.^27^

Another 2D U‐Net‐based FCN combined with an object‐based postprocessing step is proposed by Chlebus et al to segment liver and tumors; the model is supported by forest classifier step which is trained by extracted features, like shape and intensity, that help in discriminating the tumors from other structures. Other work used threefold FCN networks trained with high number of epochs, to segment the liver and tumors.^28^ The authors used two cascaded FCNs to segment liver organ in two steps, coarse then fined segmentation, which justify the higher accuracy in segmenting liver compared to tumor segmentation. On the other side, as most of the listed works used LiTS training datasets to train their models, other works used their own private datasets for model training,^29^ and fine‐tuned the model on LiTS dataset, which could affect the evaluation process and the method generalization.

## 4 DISCUSSION AND CONCLUSION

Automatic segmentation of liver and tumor from CT scans is a crucial step for preoperative planning of surgical treatment. It gives precise delineations of the liver and tumors located inside it. In this work, the proposed method utilizes the strength of deep learning FCN to extract features from many examples to predict the segmentation in test dataset; besides that, the method adds more strength by incorporating the local information in the target CT scan to refine and improve the segmentation accuracy. The proposed method proved its ability of generalization by applying it on hidden test datasets from different sources. The observable promising results on the hidden datasets can clearly suggest that the proposed method can be generalized to test other different datasets. The combination of localized level set showed the significant improvement added on the predicted segmentation, despite that the U‐Net is trained with limited number of datasets from LiTS datasets with few number of epochs (20) compared to other methods that use hundreds of training epochs.

The proposed framework starts by improving the intensity features of the liver and tumor objects. Median filtering for liver organ aims to enhance the intensity homogeneity, which helps both the FCN and level set steps to extract the accurate intensity features from this region. For tumor segmentation, the EED filtering is used to increase the contrast between the tumor tissue and other liver parenchyma. Computed tomography slices of liver envelope shown by the first row in Fig. 6 highlight the importance of EED filtering and how it increased the contrast of tumors. For liver tumor segmentation, the combined segmentation approach using deep learning and localized level set function started first by extracting the main feature, especially the contrast differences between tumors and liver parenchyma, from different training datasets to train the FCN network. This is followed by extracting the tumor intensity range of the target image using region‐based level set step, which is used to refine the initial segmentation of the FCN‐trained network. In this work, the 2D region‐based level set method is used instead of the 3D version, that is because the 2D level set segmentation performs better than the 3D in terms of curve evolution on each individual slices.

As the framework consists of two consecutive steps, liver then tumor segmentation, the potential limitation of the proposed method is that accuracy of tumor segmentation relies on the liver organ segmentation step. The segmentation of the tumors is carried inside the extracted liver envelope from first step. It could happen in some datasets that liver parenchyma has similar intensity homogeneity with tumors that impose a challenge to extract accurate liver envelope. This work assumes that all CT scans are acquired at the portal venous phase of image acquisition, in which the tumors and liver parenchyma have a clear contrast.

The proposed method demonstrated the improvement of using level set technique and the use of local information in the target image to enhance the FCN‐predicted segmentation and achieve accurate segmentation output. Based on the evaluation results, the proposed method achieved high segmentation quality in detecting liver and tumors from CT images. The proposed method succeeded to segment liver and tumors in heterogeneous CT scans from different scanners, as in IRCAD dataset, which proved its ability for generalization and be promising tool for automatic analysis of liver and its tumors in clinical routine.

## CONFLICT OF INTEREST

The authors declare that they have no conflict of interest.

## ETHICAL APPROVAL

All procedures performed in studies involving human participants were in accordance with the ethical standards of the institutional and/or national research committee and with the 1964 Helsinki declaration and its later amendments or comparable ethical standards. For this type of study, formal consent is not required.

## Informed consent

Informed consent was obtained from all individual participants included in the study.

## Notes

^1^
https://keras.io/

^2^
https://competitions.codalab.org/competitions/17094

^3^ available at http://ircad.fr/research/3d‐ircadb‐01

## References

[1] Priyadarsini S , Selvathi D . Survey on segmentation of liver from CT images. Proc 2012 IEEE Int Conf Adv Commun Control Comput Technol ICACCCT 2012; 2012:234–238.

[2] Sharma N , Aggarwal LM . Automated medical image segmentation techniques. J Med Phys/Assoc Med Phys India. 2010;35:3. 10.4103/0971-6203.58777 PMC2825001 20177565

[3] Alirr OI , Rahni AAA . Survey on liver tumor resection planning system: steps, techniques, and parameters. J Dig Imaging. 2019;33:1–20. 10.1007/s10278-019-00262-8 PMC7165210 31428898

[4] Foruzan AH , Chen YW , Zoroofi R , et al. Segmentation of liver in low‐contrast images using K‐means clustering and geodesic active contour algorithms. IEICE Trans Inf Syst. 2013;E96.D:798–807.

[5] Boas FE , Fleischmann D . CT artifacts: causes and reduction techniques. Imaging Med. 2012;4:229–240.

[6] Anter AM , Azar AT , Hassanien AE , El‐Bendary N , Elsoud MA . Automatic computer aided segmentation for liver and hepatic lesions using hybrid segmentations techniques. 2013 Fed Conf Comput Sci Inf Syst FedCSIS; 2013:193–198.

[7] Cornelis FH , Martin M , Saut O , et al. Precision of manual two‐dimensional segmentations of lung and liver metastases and its impact on tumour response assessment using RECIST 1.1. Eur Radiol Exp. 2017;1:16. 29708185 10.1186/s41747-017-0015-4 PMC5909353

[8] Long J , Shelhamer E , Darrell T . Fully convolutional networks for semantic segmentation. Proc. IEEE Comput. Soc. Conf. Comput. Vis. Pattern Recognit., vol. 07–12‐ June‐2015, IEEE Computer Society; 2015:3431–3440.

[9] Hesamian MH , Jia W , He X , Kennedy P . Deep learning techniques for medical image segmentation: achievements and challenges. J Dig Imaging. 2019;32:582–596. 10.1007/s10278-019-00227-x PMC6646484 31144149

[10] Li W , Jia F , Hu Q . Automatic segmentation of liver tumor in CT images with deep convolutional neural networks. J Comput Commun. 2015;03:146–151.

[11] Chlebus G , Schenk A , Moltz JH , van Ginneken B , Hahn HK , Meine H . Automatic liver tumor segmentation in CT with fully convolutional neural networks and object‐based postprocessing. Sci Rep. 2018;8:1–7. 30341319 10.1038/s41598-018-33860-7 PMC6195599

[12] Ronneberger O , Fischer P , Brox T . U‐net: Convolutional networks for biomedical image segmentation. Lect. Notes Comput. Sci. (including Subser. Lect. Notes Artif. Intell. Lect. Notes Bioinformatics), vol. 9351, Springer Verlag; 2015, p. 234–41.

[13] Bilic P , Christ PF , Vorontsov E , et al. The Liver Tumor Segmentation Benchmark (LiTS); 2019. 10.1016/j.media.2022.102680 PMC10631490 36481607

[14] Han X . Automatic Liver Lesion Segmentation Using A Deep Convolutional Neural Network Method; 2017.

[15] Vorontsov E , Tang A , Pal C , Kadoury S . Liver lesion segmentation informed by joint liver segmentation. Proc. ‐ Int. Symp. Biomed. Imaging, vol. 2018–April, IEEE Computer Society; 2018:1332–1335.

[16] Goryawala M , Gulec S , Bhatt R , McGoron AJ , Adjouadi M . A low‐interaction automatic 3D liver segmentation method using computed tomography for selective internal radiation therapy. Biomed Res Int. 2014;2014:12. 10.1155/2014/198015 PMC4106113 25105118

[17] Altarawneh NM , Luo S , Regan B , Sun C . A modified distance regularized level set model for liver segmentation from CT images. Sign Image Process. 2015;6:1.

[18] Chan T , Vese L . Active contours without edges. Image Process IEEE Trans. 2001;10:266–277. 10.1109/83.902291 18249617

[19] Alirr OI , Rahni AAA , Golkar E . An automated liver tumor segmentation from abdominal CT scans for hepatic surgical planning. Int J Comput Assist Radiol Surg. 2018;13:1169–1176. 29860549 10.1007/s11548-018-1801-z

[20] Alirr OI , Rahni AA . Automatic liver segmentation from ct scans using intensity analysis and level‐set active contours. J Eng Sci Technol. 2018;13:3821–3839.

[21] Christ PF , Ettlinger F , Grün F , et al. Automatic Liver and Tumor Segmentation of CT and MRI Volumes using Cascaded Fully Convolutional Neural Networks. arXiv preprint arXiv:1702.05970; 2017 Feb 20.

[22] Mendrik AM , Vonken EJ , Rutten A , Viergever MA , Van Ginneken B . Noise reduction in computed tomography scans using 3‐D anisotropic hybrid diffusion with continuous switch. IEEE Trans Med Imaging. 2009;28:1585–1594. 19783496 10.1109/TMI.2009.2022368

[23] Osher S , Fedkiw R . Level set methods: an overview and some recent results. J Comput Phys. 2001;169:463–502.

[24] Lankton S , Member S , Tannenbaum A . Localizing region‐based active contours. IEEE Trans Image Process. 2008;17:2029–2039. 18854247 10.1109/TIP.2008.2004611 PMC2796112

[25] Lankton S , Tannenbaum A . Localizing region‐based active contours. IEEE Trans Image Process. 2008;17:2029–2039. 18854247 10.1109/TIP.2008.2004611 PMC2796112

[26] Ahmad M , Ai D , Xie G , et al. Deep belief network modeling for automatic liver segmentation. IEEE Access. 2019;7:20585–20595.

[27] Gruber N , Antholzer S , Jaschke W , Kremser C , Haltmeier M . A Joint Deep Learning Approach for Automated Liver and Tumor Segmentation, Institute of Electrical and Electronics Engineers (IEEE); 2020:1–5.

[28] Yuan Y . Hierarchical convolutional‐deconvolutional neural networks for automatic liver and tumor segmentation. arXiv preprint arXiv:1710.04540; 2017 Oct 12.

[29] Chen LC , Zhu Y , Papandreou G , Schroff F , Adam H . Encoder‐decoder with atrous separable convolution for semantic image segmentation. Lect. Notes Comput. Sci. (including Subser. Lect. Notes Artif. Intell. Lect. Notes Bioinformatics), vol. 11211 LNCS, Springer Verlag; 2018:833–851.

[30] Bi L , Kim J , Kumar A , Feng D . Automatic liver lesion detection using cascaded deep residual networks. arXiv preprint arXiv:1704.02703; 2017 Apr 10.

[31] Nanda N , Kakkar P , Nagpal S . Computer‐aided segmentation of liver lesions in CT scans using cascaded convolutional neural networks and genetically optimised classifier. Arab J Sci Eng. 2019;44:4049–4062.

